# Design of a RF Switch Used in Redundant Atomic Clock Configurations

**DOI:** 10.3390/s19102331

**Published:** 2019-05-20

**Authors:** Yuqing Hou, Sangyuan Wang, Sheng Tang, Tao Zhang

**Affiliations:** School of Information Science and Technology, Northwest University, Xi’an 710127, China; houyuqin@nwu.edu.cn (Y.H.); 201731880@stumail.nwu.edu.cn (S.W.)

**Keywords:** atomic clock, redundant configuration, RF switch, switching speed

## Abstract

Atomic clocks provide frequency reference signals for communication, aerospace, satellite navigation and other systems. The redundant configuration of atomic clocks is necessary for ensuring the continuity and stability of the system. A radio frequency (RF) switch is usually used as a switching device in the switching system of the host atomic clock and the backup atomic clock. When the atomic clock fails, the switching between the host and the backup clock can be carried out quickly. Aiming at the fast switching requirements of atomic clock RF signals, this paper proposes a new series-shunt Positive Intrinsic Negative (PIN) switch design. In this paper, the evaluation of the RF switches is conducted by using the metrics of switching speed, insertion loss, isolation, return loss at on state and return loss at off state. Experimental result shows that the new PIN switch has better and more comprehensive performance metrics than the electromechanical switch, FET switch and conventional PIN switch. In particular, the switching speed is 53 ns faster than the conventional series-shunt PIN switch.

## 1. Introduction

An atomic clock is an oscillator and an auxiliary circuit which can produce high accuracy and stability standard frequency signals such as a 10 MHz sine signal [1]. They are not only used as timekeeping devices for timing systems, but also provide standard frequency signals for many devices [2]. The steady and continuous operation of atomic clocks ensures the normal operation of numerous daily systems.

Atomic clocks are used in many fields, such as electronic countermeasures, navigation, aviation, wireless communications, global positioning systems, power grid systems and so on [3,4]. The working environments of these applications are complicated. After a long period of work, the working state of the atomic clock may be affected by electromagnetic interference, component aging, temperature change and vibration interference from the surrounding environment [5]. These may cause the atomic clock to malfunction or even become damaged. In this case, the atomic clock needs to be redundantly configured [6]. When the host atomic clock fails or is damaged, the redundantly configured atomic clock can be used as a backup clock to take over the work of the host atomic clock in a timely way, thereby ensuring the normal operation of the system [7]. The redundant configuration greatly ensures the reliability of the system [8].

**Figure 1:**
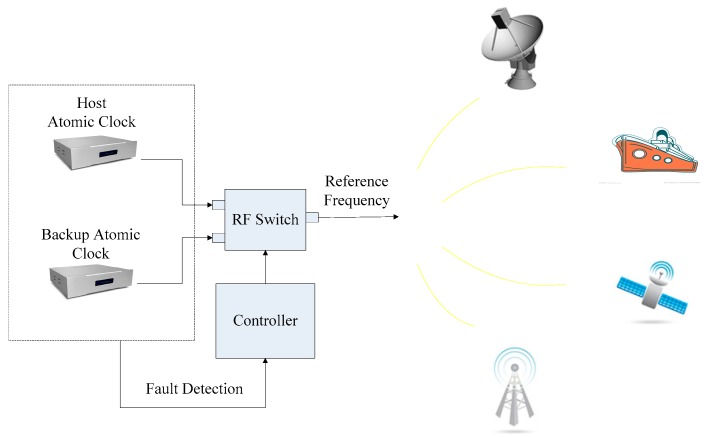
Applications of redundant atomic clock configurations.

As shown in Figure 1, the switching system of a redundant atomic clock configuration is suitable for many applications, such as radar systems, navigation systems, global positioning systems, communication systems and so on [9]. Smooth switching is one of the core technologies in the redundant configuration of the atomic clocks [10]. In order to achieve smooth switching between the host atomic clock and the backup atomic clock, the following two requirements must be met: on the one hand, so as to ensure the frequency difference and phase difference between the two signals are as small as possible, the phase and frequency of two signals need to be adjusted before switching. On the other hand, the “switching” action of smooth atomic clock switching is done by a switch. The switching speed of the switch should be as fast as possible, and the isolation should be high enough to minimize the phase jump caused by the switching. The mutual influence between the two signals should be reduced and the original technical metrics can be maintained. The RF switch is one of the core components of the system which is usually used as a switching device for the switching system of the host and the backup clock [11]. According to the working condition of the atomic clock, the user can select the atomic clock to be used by controlling the on/off of the RF switch and complete the switching between the host atomic clock and the backup atomic clock. Therefore, an RF switch with fast switching speed and high isolation is an indispensable part in the switching system of the host and backup clocks.

In order to meet the requirements of the smooth switching of the RF signal by atomic clocks, this paper analyzes the technical performance metrics of different RF switches. By comparing their switching speed, insertion loss, isolation, return loss and other technical performance metrics, this paper proposes a new PIN series-shunt switch as the switch device for the switching system of atomic clock redundant configurations. Compared with the electromechanical switch, the FET switch and the conventional PIN switch, the new PIN switch has better performance and is more suitable for the switching system of atomic clocks. This paper consists of five parts. The first part mainly describes the research object and research background of this paper. The second part explains the basic working principle of common RF switches, and analyzes their technical metrics to select the suitable RF switch for the smooth switching of atomic clocks. The third part focuses on the solid-state switch, then describes the working principle of PIN diodes. A new PIN series-shunt switch is designed by comparing and analyzing the advantages and disadvantages of the conventional PIN switch. The fourth part designs and builds an experimental platform to verify the effectiveness of the proposed PIN switch design. The final part summarizes the main work and contributions of this paper.

## 2. Comparison of Different RF Switches

**Figure 2:**
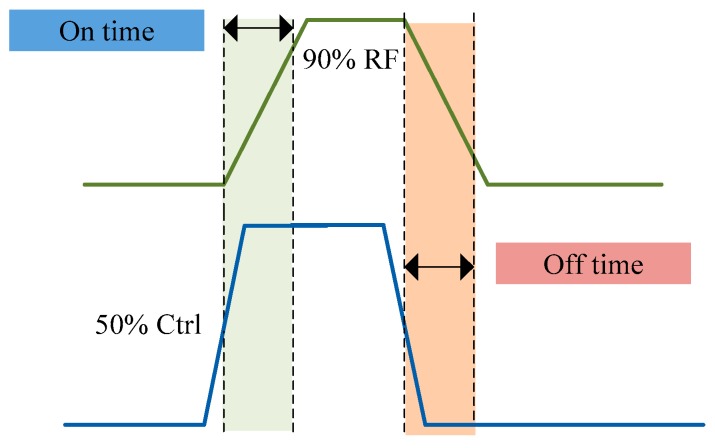
The definition of switching speed.

To evaluate the performance of a RF switch, the commonly used technical performance metrics include insertion loss, isolation, return loss and switching speed [12]. Insertion loss refers to the loss of load power due to the insertion of components or devices somewhere in the transmission system. For a RF switch, it is expressed as the ratio of the received power on the load before insertion to the received power on the same load after insertion in decibels [13,14]. Isolation refers to the ratio of the power transmitted from the switch in the off state to the load when the switch is in the on state. It reflects the degree of mutual interference among the signals of the switch [15,16]. Return loss is the loss of power in the signal returned/reflected by a discontinuity in a transmission line or optical fiber. This discontinuity can be a mismatch with the terminating load or with a device inserted in the line. It is usually expressed as a ratio in decibels [17]. Switching speed is an important technical parameter of the RF switch [18], which reflects the time needed to switch the on-off state of the RF switch [19]. Switching speed is defined as the time required to switch port state from “ON” to “OFF” or from “OFF” to “ON”. It is mainly described by the following definition: On time is measured from the 50% level of the input control signal to the 90% point of the square-law detected RF power when the unit is switched from full OFF to full ON. Off time is measured from the 50% level of the input control signal to the 10% point of the square-law detected RF power when the unit is switched from full ON to full OFF. As shown in Figure 2:

In a redundant atomic clock configuration, the selected RF switch should take into account the switching speed, insertion loss, isolation and other technical metrics. When the host atomic clock and the backup atomic clock are switched, a phase jump of their reference frequency signal is caused. The phase jump of the reference frequency signal will further deteriorate the system performance. For example, for a navigation positioning system with high precision requirements, an error on the order of nanoseconds may cause measurement errors of several tens of meters. Therefore, in order to ensure the continuity and stability of the reference frequency signals of the host clock and the backup clock before and after switching, the switching speed should be considered as a primary indicator.

Different RF switches have different mechanical and electrical structures, so their switching speed, isolation, insertion loss, return loss and other technical metrics are also different [20]. At present, common RF switches are mainly divided into two categories: electromechanical switches and solid-state switches [21,22]. Depending on the different components, solid-state switches can be roughly divided into FET switches and PIN switches. These switches have different switching characteristics and should be applied to different occasions. Electromechanical switches have the performance metrics of high isolation, low insertion loss, and wide bandwidth [23,24,25,26]. However, their disadvantages are also very obvious. Firstly, the electromechanical switches use a mechanical structure, so it will take a long time to turn on or turn off. Secondly, the lifetime of electromechanical switches is short and affected by the number of times the internal reed used [27]. Their service life is about several hundred thousand times. Thirdly, the electromechanical switch will generate an arc when switching, which may damage the contact and reduce the reliability of the RF switch.

Compared with the electromechanical switch, the solid-state switch is completely based on semiconductor characteristics. It has the characteristics of fast switching speed, long lifetime, and high reliability [28,29,30,31].

In practical engineering applications, the ON time of different RF switches is often different from the OFF time. In this paper, the switching speed is specially referred to the ON time. For example, the switching speeds of Agilent 8765B electromechanical switch and Mini-Circuits ZMSW-1211 solid-state switch are measured as shown in Table 1.

**Figure 3:**
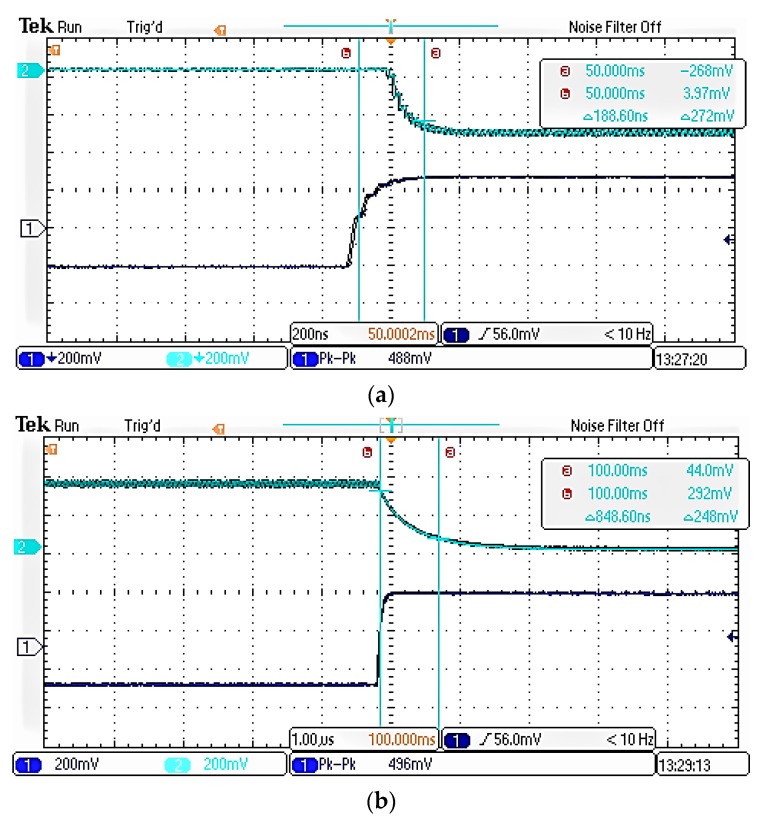

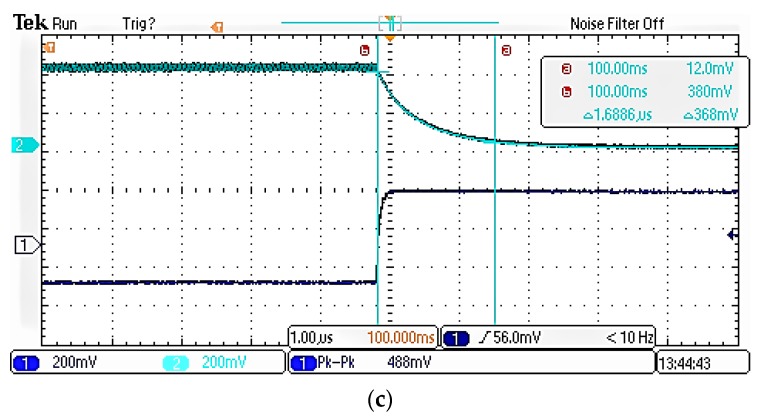
Switching speed with different coupling capacitance values (**a**) 100 pf (**b**) 500 pf and. (**c**) 1000 pf.

**Figure 4:**
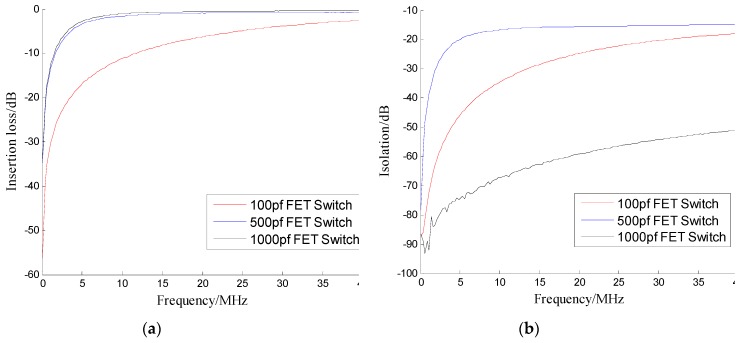
FET switch with different coupling capacitance values: (**a**) Insertion loss (**b**) Isolation.

As shown above, the switching speed of the solid state switch is much better than that of the electromechanical switch, so the solid-state switch should be the best choice for the switching system of an atomic clock redundant configuration. Compared with other solid-state switches, the FET switch and the PIN switch have the advantages of faster response speed, and their circuit structures are simple [9,32]. Compared with the FET switch, the PIN switch is more suitable for this system. On the one hand, the control circuits for voltage-controlled FET devices often have a fixed RC delay that limits their switching speed [22]. For this switching system, the switch is directly controlled by the MCU and many FET switches are designed by depletion mode FET which requires negative voltage conduction. Although it can generate negative logic voltages with TTL/CMOS circuits, this undoubtedly increases the complexity of the circuit and the time-delay. On the other hand, the insertion loss and isolation performance of the PIN switch are considered to be superior to conventional FET switches [33]. To verify this point, we measured a FET switch product (AS179-92LF). The experimental results are shown in the following Figure 3 and Figure 4.

Just as shown in Table 2, choosing the appropriate coupling capacitor can make the switching speed of the FET switch faster, but at the same time, the insertion loss, isolation and working bandwidth of the FET switch will become worse, so the comprehensive performance of the FET switch can’t meet the expectations for this switching system. Because of its flexible design, fast switching speed, high power handling and low cost, the PIN diode is used in many applications [34]. Therefore, this paper will design a solid-state switch as the switch of atomic clocks redundant configuration and specifically select the PIN switch as the design reference.

## 3. Design of the PIN Switch

### 3.1. Principle of PIN Diode

The PIN diode is based on common PN diodes. Unlike conventional PN diodes, The PIN diode adds a thin layer of low-doped intrinsic material between the positive pole and the negative pole. The I-layer can reduce the interstage capacitance of the diode and increase the breakdown voltage [35].

**Figure 5:**
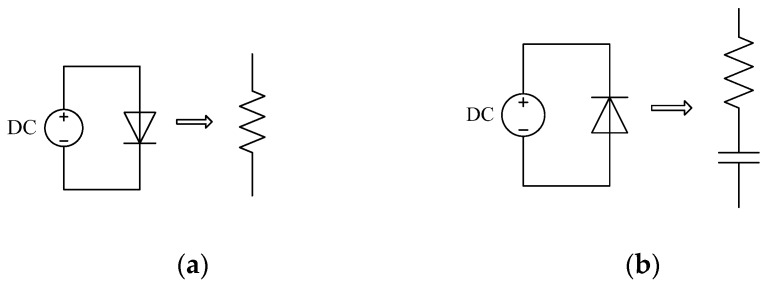
(**a**) Forward bias model of PIN diode and (**b**) Reverse bias model of PIN diode.

When the PIN diode is forward biased, the PIN diode is turned ‘ON’ and the diode presents a low resistance state [36]. It can be considered as a small resistance, with a resistance value between 0.1 Ohm and 10 Ohm, just as shown in Figure 5a. When the PIN diode is reversed, the PIN diode is turned ‘OFF’. And the diode presents a high capacitance state [37]. It can be seen as a large resistor in series with a capacitor, just as shown in Figure 5b. The resistance is generally between 0.1 PF and 10PF. The PIN diode used for this design is a Skyworks smp1340-079LF, which has a resistance of 1 Ohm and a junction capacitance of 0.3 pf. In addition, minority carrier lifetime is also an important parameter [38]. The switching speed of the PIN switch is related to minority carrier lifetime of the PIN diode. Switching speed is negatively correlated with the minority carrier lifetime [39]. The carrier lifetime of the smp1340-079LF is 100 ns.

### 3.2. Conventional PIN Switch

The PIN switch circuit has three basic structural forms in design, which are the series type, the shunt type, and the series-shunt type. Each of the three switches has its own advantages and disadvantages. Then, the three circuit structures will be theoretically analyzed and measured.

**Figure 6:**
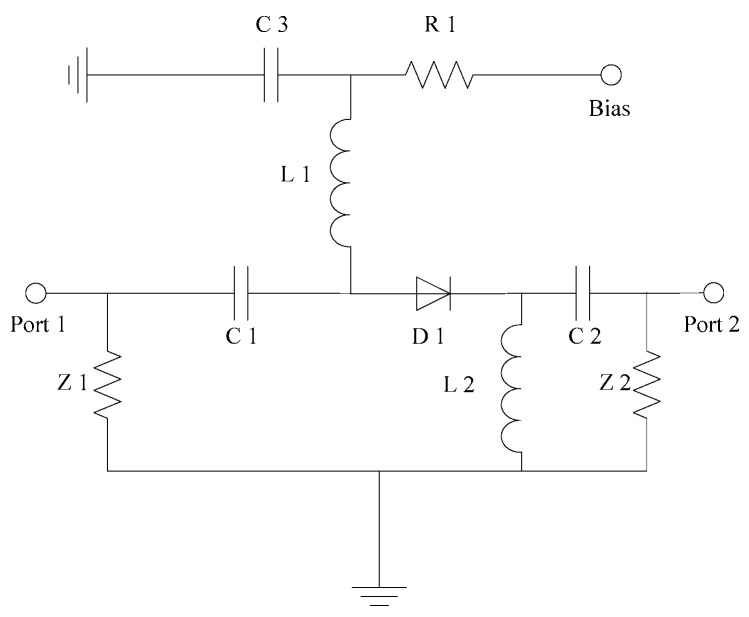
Circuit schematic of the series PIN switch.

Figure 6 shows a schematic of a series PIN switch circuit. C1 and C2 are DC blocking capacitors. C3 is a filter capacitor of the bias circuit. L1 and L2 are RF chokes. And when the bias terminal is under positive voltage, the D1 is forward biased and the switch is turned on. When the bias terminal is under negative voltage, the diode D1 is reversed and the switch is turned off. The series switch has high isolation, but the disadvantage is that the insertion loss is large.

(1) $$I_{L}=-20\;\log_{10}(1+R_{S}/2Z_{0})$$

Equation (1) is the calculation formula for the insertion loss of the series SPST switch. For multi-throw switches, the insertion loss will increase due to the impedance mismatch caused by the capacitance of the PIN diode in the branch:

(2) $$I_{SO}=-10\;\log_{10}[1+{\left(4\pi fC_{T}Z_{0}\right)}^{-2}]$$

Equation (2) is the calculation formula for the isolation of the series SPST switch. It shows that the switch isolation is determined by the diode reverse bias capacitance.

**Figure 7:**
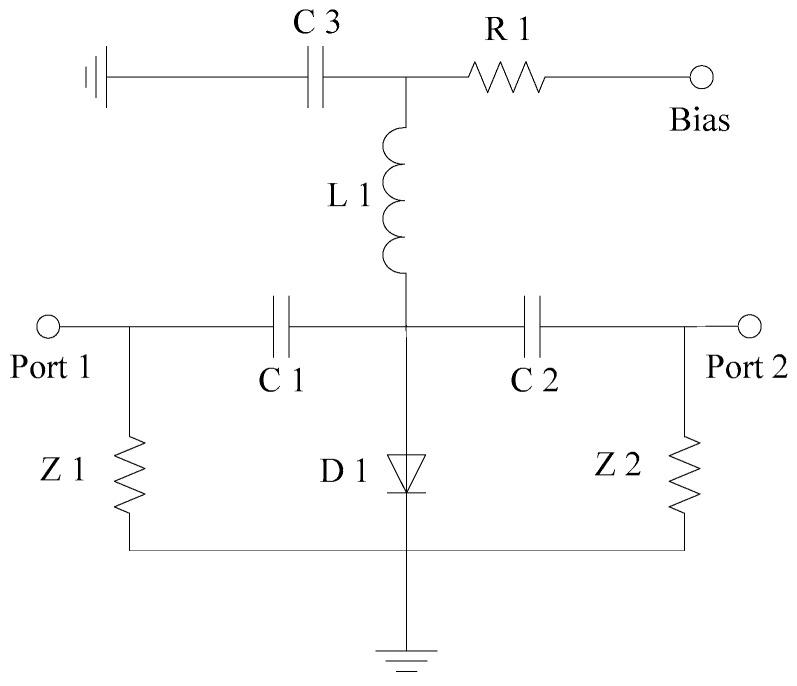
Circuit schematic of the shunt PIN switch.

Figure 7 shows a schematic of a shunt PIN switch circuit. When the bias terminal is under positive voltage, D1 is forward biased and the switch is turned off. When the bias terminal is under negative voltage, D1 is reversed and the switch is turned on. Compared with the series PIN switch, the shunt switch has less insertion loss but lower isolation:

(3) $$I_{L}=-10\;\log_{10}[1+{\left(\pi fC_{T}Z_{0}\right)}^{-2}]$$

Equation (3) is the calculation formula for the insertion loss of the shut SPST switch. Compared with the Equation (1), it clearly shows that the insertion loss of the shunt PIN switch is less than the insertion loss of the series PIN switch for the same kind of PIN diode:

(4) $$I_{SO}=-20\;\log_{10}(1+Z_{0}/2R_{S})$$

Equation (4) is the calculation formula for the isolation of the shunt SPST switch. Compared with the Equation (2), it is obvious that with the same kind of PIN diodes, the isolation of the shunt PIN switch is less than the isolation of the series PIN switch.

**Figure 8:**
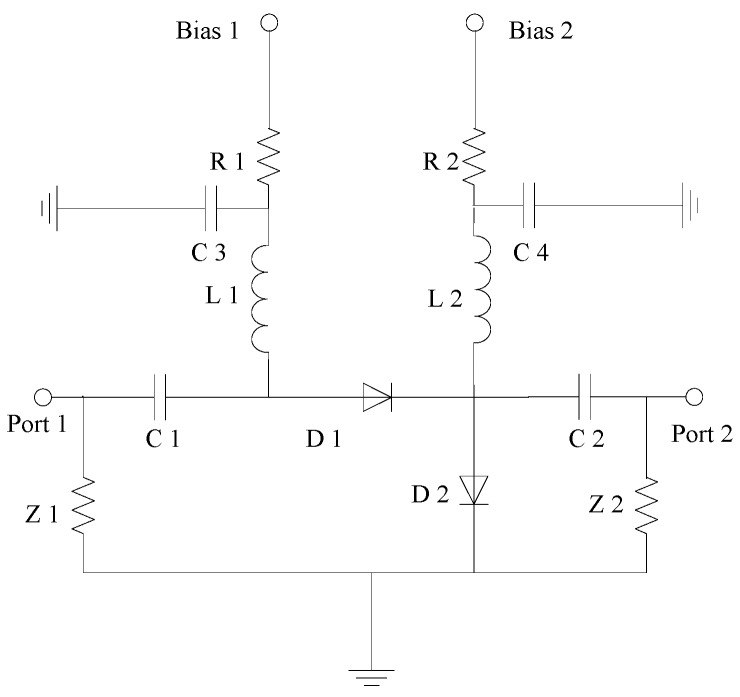
Circuit schematic of the series-shunt PIN switch.

In order to combine the advantages of the series PIN switch with the shunt PIN switch, more serial-shunt PIN switches are used in the design. The two bias ports respectively control the on and off of D1 and D2. When Bias1 is high level and Bias2 is low level, D1 is forward biased and D2 is reverse biased. The switch is in the on state. When Bias1 is Low level as well as Bias2 is high level, D1 is forward biased and D2 is reverse biased. The switch is in the off state. The circuit schematic of the series-shunt PIN switch is shown in Figure 8.

We measured the transmission of 10MHz RF signals for three switches under 50 Ohm load. The specific measurement data is shown in Table 3. It is clearly shown in Table 3 that when a single diode is used, the isolation of the series switch is higher than that of the shunt switch. And the insertion loss of the shunt switch is lower than that of the series switch. Consequently, the series-shunt switch integrates the advantages of the series switch and the shunt switch.

### 3.3. Design of the Improved Series-Shunt PIN Switches

**Figure 9:**
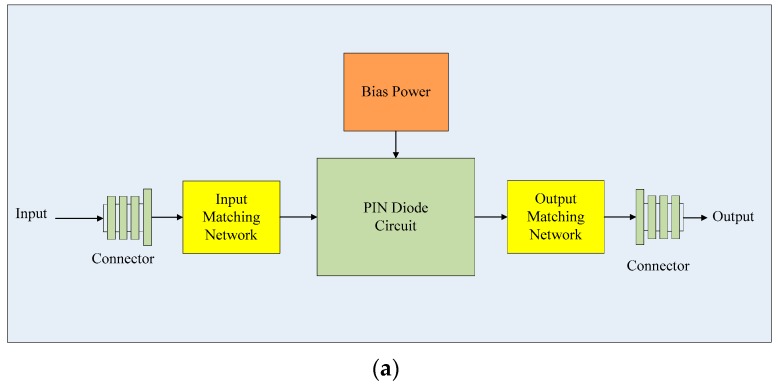

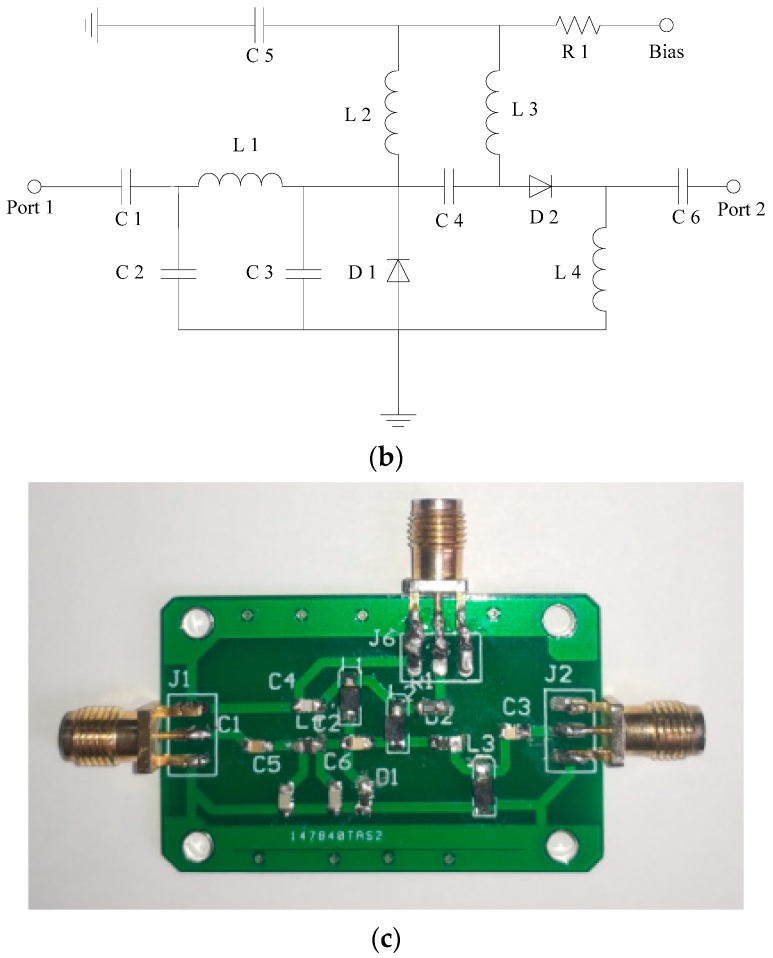
Series-shunt PIN switch: (**a**) block diagram of improved PIN switch (**b**) analog of the circuit schematic and (**c**) final design.

As shown in Figure 9, the basic principle of the improved series-shunt PIN switch is similar to that of the conventional series-shunt PIN switch. On the basis of the conventional series-shunt PIN switch, the new switching circuit is connected in series with a separated capacitor C4 between D1 and D2. The diode D1 and D2 can be controlled simultaneously with only one bias terminal, and the DC bias circuits of D1 and D2 are independent of each other. Compared with the two-terminal control diode layout, the single-terminal control can ensure the synchronization of the two PIN diodes to the most extent.

We added a Π-type matching network composed of L1, C2 and C3 to the input. The matching network is often used to select the band in the transceiver switching circuit of the wireless communication system. The improved series-shunt PIN switch designed is used in the redundant switching system of atomic clocks, so the designed working frequency band is 5 MHz–100 MHz.

For the atomic clock redundant configuration, a host atomic clock and a backup atomic clock are usually required. Therefore, a Single-pole Double-throw (SPDT) switch can be used to accomplish such switching tasks. Designing a SPDT switch requires simply adding an input port based on the proposed switch. Second thing to notice, there may be interference between the two input signals. So when designing the layout of PCB, the position of the two input ports should be kept as far as possible. This can effectively reduce signal interference and improve the isolation of the switch.

## 4. Experiment

In order to verify the effectiveness of the new PIN switch design scheme proposed in this paper, we designed and built a test platform. The experimental equipment used includes a PRS10 rubidium atomic clock, a SDG1062X signal generator, a Tektronix DPO 2022B digital oscilloscope, a DEVISER NA7632A vector network analyzer and a detector.

### 4.1. Measurement of Switching Speed

**Figure 10:**
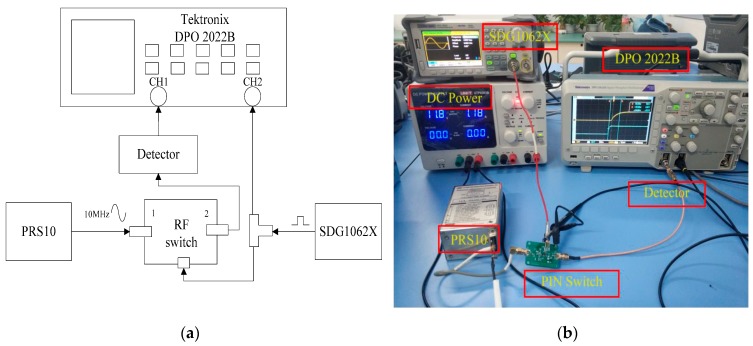
Platform for testing the switching speed of RF switches: (**a**) Block diagram of the experimental platform and (**b**) Photograph of the experimental platform.

**Figure 11:**
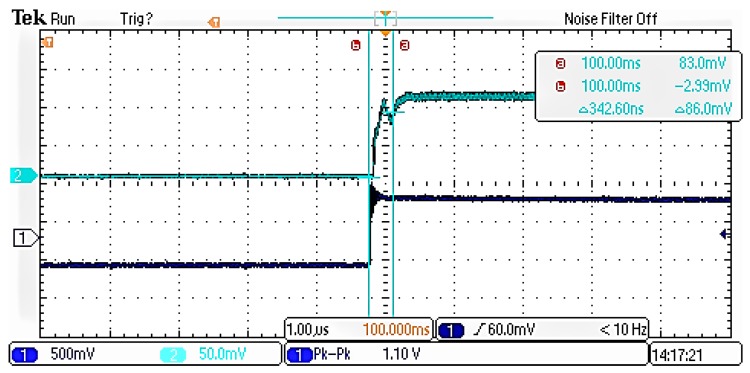
Switching speed of the improved series-shunt switch.

As shown in Figure 10 the switching speed measurement platform includes an atomic clock, a signal generator, a detector and a digital oscilloscope. The signal generator inputs a square wave signal to the switch control terminal and controls the turn-on and turn-off of the RF switch. The atomic clock inputs 10 MHz standard sine signal to the RF switch, and the sine signal is output to the detector through the radio frequency switch. Finally, the digital oscilloscope is used to observe the waveform. The switching speed can be directly observed by the oscilloscope just as shown in Figure 11.

### 4.2. Measurement of Other Technical Metrics

**Figure 12:**
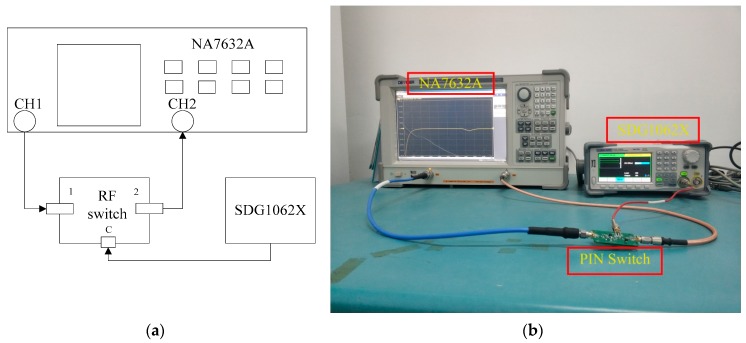
Experimental platform for testing the other technical metrics of RF switches: (**a**) Block diagram of the experimental platform and (**b**) Photograph of the experimental platform.

As shown in Figure 12, the platform for measuring other performance metrics of RF switches is composed of the switch to be measured, a signal generator and a vector network analyzer. The PIN switch input is connected to the vector network analyzer port1. The output is connected to port2. And the vector network analyzer measures the S parameter of the RF switch. When the switch is turned on, S(2,1) is measured as the insertion loss of the switch. When the switch is turned off, S(2,1) is measured as the isolation of the switch. The formula is as follows:

(5) $$S(2,1)=10\;\log_{10}(P_{output}/P_{input})$$

When the switch is turned on, S(1,1) is measured as the return loss at on state. When the switch is turned off, S(1,1) is measured as the return loss at off state. The formula is as follows:

(6) $$R_{L}=S(1,1)=10\;\log_{10}P_{ref}/P_{input}$$

We measured the insertion loss, isolation, return loss, and switching speed of the improved series-shunt PIN switch and compared it with three conventional PIN switches.

**Figure 13:**
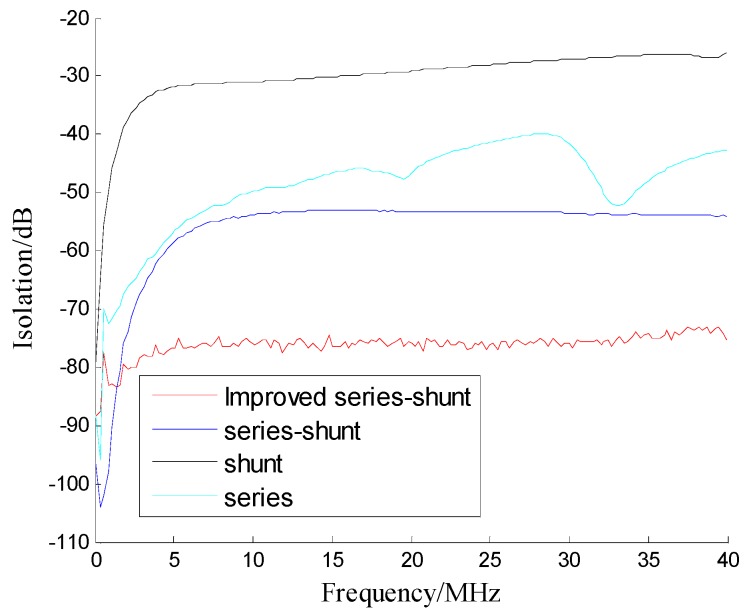
Isolation performance of conventional and improved switches.

**Figure 14:**
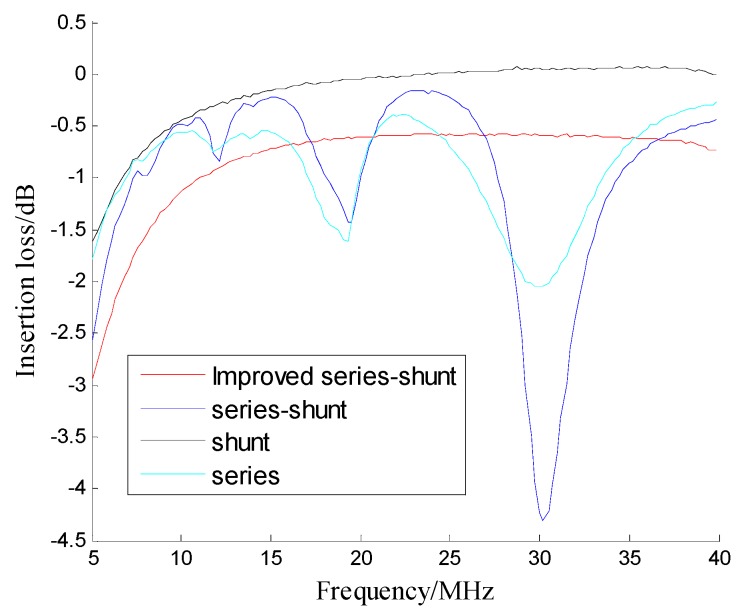
Insertion loss performance of conventional and improved switches.

The experimental results are as follows:

As Shown in Figure 13 and Figure 14, the improved series-shunt PIN switch can increase the isolation of a 10 MHz signal by 23 dB, the switching speed by 53 ns, and the insertion loss is slightly worse than that of a conventional PIN switch. In order to increase the switch isolation, more PIN diodes can be connected in the switch circuit. Table 4 shows the technical performance of the conventional PIN switch and our improved series-shunt PIN switch.

According to Table 4, in the comprehensive comparison of key technical metrics for RF switches, the improved series-shunt PIN switch is superior to the conventional PIN switch, so that the improved series-shunt PIN switch is more suitable for the redundant atomic clock configuration than the conventional PIN switch.

## 5. Conclusions

This paper presents a RF switch used in a redundant configuration of 10 MHz atomic clocks. Firstly, we analyzed from theoretical and experimental perspectives the performance of the electromechanical switch, the FET switch and the PIN switch. Consequently, we verified that PIN switches are more suitable for the switching system which is used in a redundant atomic clock configuration. Then based on the principle of PIN diodes, we compared the three conventional connection modes of PIN switches. Through theoretical and experimental verification, the process proves that the series-shunt switch has the best performance. On this basis, we design an improved series-shunt PIN switch. The experimental results show that compared with the conventional series-shunt PIN switches, the improved series-shunt PIN switch can significantly improve the key technical metrics of performance such as isolation, return loss and switching speed, so it is the most suitable RF switch for a redundant configuration of atomic clocks.

## Figures and Tables

**Table 1:** The statistics of the measurement result.

Switch Types	Switching Speed
Electromechanical Switch (Agilent 8765B, e.g.,)	26.20 ms
Solid-state Switch (Mini-Circuits ZMSW-1211, e.g.,)	4.00 us

**Table 2:** Performance comparison of FET switches by 10MHz signal.

Capacitance	Switching Speed	Insertion Loss	Isolation
100 pf	188.60 ns	−11.12 dB	−34.36 dB
500 pf	848.60 ns	−1.23 dB	−16.15 dB
1000 pf	1688.60ns	−0.78 dB	−64.83 dB

**Table 3:** Comparison of insertion loss and isolation for different Switches.

Switch Types	Insertion Loss (dB)	Isolation (dB)
Series Switch	−0.570	−49.730
Shunt Switch	−0.443	−31.782
Conventional Series-shunt Switch	−0.482	−53.826

**Table 4:** Performance of different switches.

Switch Types	Insertion Loss (dB)	Isolation (dB)	Switching Speed (ns)	Return Loss at on-State (dB)	Return Loss at Off-State (dB)
Series switch	–0.57	–49.73	331.20	–0.65	–11.54
Shunt switch	–0.44	–31.78	691.20	–0.45	–9.10
Conventional Series-shunt switch	–0.48	–53.83	396.00	–0.45	–17.02
Improved Series-shunt switch	–1.14	–76.33	342.60	–0.15	–6.89
